# Supplementary material for: Trends in the Number of Behavioural Theory-Based Healthy Eating Interventions Inclusive of Dietitians/Nutritionists in 2000–2020
Source: Nutrients. 2021 Nov 20;13(11):4161. doi: 10.3390/nu13114161 (PMC8623843; doi:10.3390/nu13114161)
Supplement: Supplementary file 1 [file nutrients-13-04161-s001.zip › supplementary Tables.pdf]

Table S1. Complete search strategy in Medline.

1. (THEOR* adj2 (COGNITIVE or TRANSTHEORETICAL or TRANS THEORETICAL or PLANNED or CONTROL or SELF-DETERMINATION)).tw.
2. (MODEL* adj2 (TRANSTHEORETICAL or TRANS THEORETICAL)).tw.
3. (Model* adj2 Information Motivation Behavio* Skill*).tw.
4. SOCIAL COGNITIVE THEOR*.tw.
5. SOCIAL LEARNING THEOR*.tw.
6. Health Action Process Approach.tw.
7. (BEHAVIO* adj2 (THEOR* or TECHNIQUE*)).tw.
8. health belief model*.tw.
9. Diet, Healthy/
10. healthy people programs/
11. weight reduction programs/
12. healthy lifestyle/
13. ((NUTRITION* or DIET* or HEALTHY EATING) adj1 (KNOWLEDGE or GUIDELINE* or INTERVENTION* or EDUCATION or PROGRAM* or INITIATIVE* or STRATEG* or CAMPAIGN* or ADVICE* or COACH* or COUNSEL*)).tw.
14. exp Beverages/
15. Bread/
16. exp Candy/
17. Chocolate/
18. Fruit/
19. Vegetables/
20. Meat/
21. Nuts/
22. Salads/
23. exp Seeds/
24. exp Dairy Products/
25. exp dietary carbohydrates/
26. exp dietary fats/
27. exp dietary fiber/
28. exp dietary proteins/
29. exp Eggs/
30. Fast foods/
31. (FOOD* adj1 (TAKEAWAY or CONVENIEN* or FAST or DISCRETIONARY)).tw.
32. (FOOD* adj1 (ENERGY-DENSE or NUTRIENT-POOR or ULTRA-PROCESSED or PROCESSED)).tw.
33. FRUIT*.tw.
34. VEGETABLE*.tw.
35. GRAIN*.tw.
36. (BREAD or CEREAL*).tw.
37. MEAT*.tw.
38. (NUT* or SEED*).tw.
39. DAIRY.tw.
40. MILK.tw.
41. EGG*.tw.
42. FOOD GROUP*.tw.
43. ((SUGAR* or SWEET* or ENERGY) adj2 (BEVERAGE* or DRINK*)).tw.
44. (DIETARY adj1 (CARBOHYDRATE* or FAT* or FIBRE* or PROTEIN*)).tw.
45. Portion Size/
46. Serving Size/
47. Food Preferences/
48. (EATING adj2 (BEHAVIO* or HABIT* or PRACTICE*)).tw.
49. (SIZE* adj1 (PORTION or SERV*)).tw.
50. (PREPAR* adj2 (FOOD* or MEAL*)).tw.
51. (OVEREAT* or OVER-EAT*).tw.
52. or/1-8
53. or/9-51
54. 52 and 53
55. limit 54 to (english language and yr="2000 -Current")
56. limit 55 to humans

Table S2. Summary of Studies.

Author (Ref number)	Year	Dietitian/Nutritionist	Country	Dietary behaviour	Theory	BCTs taxonomy
Okayama et al [11]	2019	No	Japan	Vitamin D food sources	Transtheoretical model	No
Gregorio-Pascual et al [12]	2019	No	USA	Sugar-sweetened beverages	Theory of planned behaviour	No
Ainscough et al [13]	2019	Yes	Ireland	Healthy eating	Control theory<br>Social cognitive theory<br>COM-B model	Yes
Wright et al [14]	2019	Yes	USA	Fruits and/or vegetables	Health belief model	No
White et al [15]	2019	Yes	USA	Cooking skills & Healthy eating	Social cognitive theory	No
Smith et al [16]	2019	Yes	USA	Dairy products	Social cognitive theory	No
Shirazi et al [17]	2019	No	Iran	Healthy eating	Social cognitive theory	No
Rahimdel et al [18]	2019	Yes	Iran	Salt intake	Theory of planned behaviour	No
Pope et al [19]	2019	No	USA	Fruits and/or vegetables<br>Calorie intake	Social cognitive theory<br>Self-determination theory	No
Poole et al [20]	2019	Yes	USA	Healthy eating	Social cognitive theory	No
Parandeh et al [21]	2019	No	Iran	Calcium intake	Health belief model	No
Ovrebo et al [22]	2019	Yes	Norway	Fruits and/or vegetables	Social cognitive theory	No
Muzaffar et al [23]	2019	Yes	USA	Healthy eating	Social cognitive theory<br>Stage of change model ie from Transtheoretical model	No
Meurer et al [24]	2019	Yes	Brazil	Healthy eating	Social cognitive theory	No
Leme et al [25]	2019	Yes	Brazil	Healthy eating	Social cognitive theory	No
Kulik et al [26]	2019	No	USA	Healthy eating	Social cognitive theory	No
Koch et al [27]	2019	Yes	USA	Healthy eating	Social cognitive theory<br>Self-determination theory	No
Keshani et al [28]	2019	Yes	Iran	Healthy eating	Health belief model	No
Jung et al [29]	2019	No	USA	Healthy eating	Social cognitive theory	No
Heikkila et al [30]	2019	Yes	Finland	Healthy eating	Self-Determination Theory	No
Hammersley et al [31]	2019	Yes	Australia	Healthy eating	Social cognitive theory	No
Ghaffari et al [32]	2019	No	Iran	Fruits and/or vegetables	Social cognitive theory	No
Augustin et al [33]	2019	Yes	USA	Healthy eating	Theory of planned behaviour<br>Social ecological theory	No
Alami et al [34]	2019	No	Iran	Iron and Vitamin D supplements	Theory of planned behaviour	No
Worawong et al [35]	2018	No	USA	Fruits and/or vegetables &<br><10% fat	Self-regulation theory<br>Self-determination theory	No

van Doorn-van Atten et al [36]	2018	Yes	Netherlands	Healthy eating	Control theory	Yes
Park et al [37]	2018	Yes	USA	Healthy eating	Social cognitive theory	No
Overby et al [38]	2018	Yes	USA	Healthy eating	Health belief model	No
Myers et al [39]	2018	Yes	USA	Healthy eating	Social cognitive theory	No
Lion et al [40]	2018	No	Nigeria	Green leafy vegetables and bouillon cubes	Theory of planned behaviour	Yes

**Author (Ref number)**	**Year**	**Dietitian/Nutritionist**	**Country**	**Dietary behaviour**	**Theory**	**BCTs taxonomy**
Lane et al [41]	2018	Yes	USA	Sugar-sweetened beverages	Theory of planned behaviour	No
Lakshman et al [42]	2018	No	UK	Dairy products	Social cognitive theory	Yes
Knowlden et al [43] [84][103]	2018	No	USA	Fruits and/or vegetables & Sugar-sweetened beverages	Social cognitive theory	No
Hatami et al [44]	2018	Yes	Iran	Healthy eating	Health belief model	No
Ellis et al [45]	2018	Yes	USA	Healthy eating	Social cognitive theory	No
Amirzadeh Iranagh et al [46]	2018	No	Iran	Healthy eating	Health belief model	No
Ahmadi et al [47]	2018	No	Iran	Discretionary foods (fast food)	Social cognitive theory	No
Wentzel-Viljoen et al [48]	2017	Yes	South African	Salt intake	Theory of reasoned action	No
Sharma et al [49]	2017	Yes	USA	Fruits and/or vegetables	Theory of planned behaviour<br>Social cognitive theory	No
Rosario et al [50]	2017	No	Portugal	Healthy eating	Health promotion model<br>Social cognitive theory	No
Nerud et al [51]	2017	No	USA	Healthy eating	Social cognitive theory	No
Nakamura et al [52]	2017	No	Japan	Fruits and/or vegetables	Social cognitive theory<br>Theory of planned behaviour<br>Health belief model	No
Lin et al [53]	2017	No	Iran	Fruits and/or vegetables	Health action process approach	Yes
Laramee et al [54]	2017	Yes	Canada	Decreasing restrictive dietary behaviors	Theory of planned behaviour	No
Kehm et al [55]	2017	No	USA	Fruits and/or vegetables<br>Healthy eating	Health belief model	No
Kalkim et al [56]	2017	No	Turkey	Calcium intake	Health belief model	No
Heeren et al [57]	2017	No	South African	Fruits and/or vegetables<br>Fat intake	Social cognitive theory<br>Theory of planned behaviour	No
Duan et al [58]	2017	No	China	Fruits and/or vegetables	Health action process approach	Yes

Dinkel et al [59]	2017	No	USA	Healthy eating	Transtheoretical model<br>Social cognitive theory	No
de Bruijn et al [60]	2017	No	Netherlands	Fruits and/or vegetables<br>Discretionary foods	Theory of planned behaviour	No
Carfora et al [61]	2017	No	Italy	Healthy eating (decreasing processed meat)	Theory of planned behaviour	Yes
Burgermaster et al [62]	2017	Yes	USA	Healthy eating	Social cognitive theory<br>Self-determination theory	No
Brookie et al [63]	2017	Yes	New Zealand	Fruits and/or vegetables	Health action process approach	Yes
Bird et al [64]	2017	Yes	USA	Healthy eating on a budget	Theory of planned behaviour	No
Araban et al [65]	2017	No	Iran	Calorie, iron, folic acid intake	Health belief model	No
**Author (Ref number)**	**Year**	**Dietitian/Nutritionist**	**Country**	**Dietary behaviour**	**Theory**	**BCTs taxonomy**
Zoellner et al [66]	2016	Yes	USA	Sugar-sweetened beverages	Theory of planned behaviour	No
Yoong et al [67]	2016	Yes	Australia	Healthy eating	Theory of planned behaviour	No
Tavakoli et al [68]	2016	No	Iran	Healthy eating	Health belief model	No
Taghdisi et al [69]	2016	No	Iran	Fruits and/or vegetables	Theory of planned behaviour	No
Storm et al [70]	2016	No	Germany and Netherlands	Fruits and/or vegetables	Health action process approach	No
Shobeiri et al [71]	2016	No	Iran	Calcium intake	Health belief model	No
Schultz et al [72]	2016	Yes	USA	Healthy eating	Social ecological model	No
Roberts-Gray et al [73]	2016	Yes	USA	Healthy eating	Social cognitive theory<br>Theory of reasoned action	Yes
Plaete et al [74]	2016	No	Belgium	Fruits and/or vegetables	Self-regulation theory<br>Health action process approach	Yes
Mulualem et al [75]	2016	Yes	Ethiopia	Child feeding	Health belief model	No
Mirzaei et al [76]	2016	No	Iran	Reducing hot tea consumption	Theory of planned behaviour	No
Miller et al [77]	2016	Yes	USA	Healthy eating/cooking	Social cognitive theory	No
Mauriello et al [78]	2016	No	USA	Fruits and/or vegetables	Transtheoretical model	No
Lin et al [79]	2016	No	China	Discretionary foods	Social cognitive theory	No
Lein et al [80]	2016	Yes	USA	Calcium intake<br>Vitamin D food sources	Health belief model	No
Lawlor et al [81]	2016	No	UK	Healthy eating	Social cognitive theory	No

Kocken et al [82]	2016	Yes	Netherlands	Healthy eating	Theory of planned behaviour	No
Ko et al [83]	2016	No	USA	Fruits and/or vegetables<br>Healthy eating	Social cognitive theory	No
Knol et al [85]	2016	Yes	USA	Healthy eating	Social cognitive theory	No
Iranagh et al [86]	2016	No	Iran	Healthy eating	Health belief model	No
Goodman et al [87]	2016	Yes	Canada	Vitamin D food sources	Theory of planned behaviour	No
Girelli et al [88]	2016	No	Italy	Healthy eating	Self-determination theory	No
Appleton [89]	2016	No	UK	Fruits and/or vegetables	Theory of planned behaviour	No
Alidosti et al [90]	2016	No	Iran	Healthy eating	Health belief model	No
Ahn et al [91]	2016	No	USA	Fruits and/or vegetables	Social cognitive theory	No
Triador et al [92]	2015	Yes	Canada	Fruits and/or vegetables	Social cognitive theory	No
Tavassoli et al [93]	2015	No	Iran	Fruits and/or vegetables	Health belief model	No
Tariku et al [94]	2015	Yes	Ethiopia	Infant feeding practices	Health belief model	No
Shin et al [95]	2015	Yes	USA	Healthy eating	Social cognitive theory	No
Savoie et al [96]	2015	Yes	USA	Healthy eating	Theory of planned behaviour	No
Pooreh et al [97]	2015	No	Iran	Healthy eating	Theory of planned behaviour	No
Monroe et al [98]	2015	Yes	USA	Green eating behaviours	Transtheoretical model	No
Menezes et al [99]	2015	Yes	Brazil	Healthy eating	Transtheoretical model	No

**Author (Ref number)**	**Year**	**Dietitian/Nutritionist**	**Country**	**Dietary behaviour**	**Theory**	**BCTs taxonomy**
Marchetti et al [100]	2015	No	Italy	Healthy eating	Transtheoretical model<br>Social cognitive theory<br>Self-determination theory	No
Leblanc et al [101] [102]	2015	Yes	Canada	Mediterranean Diet	Self-determination theory	No
Khoramabadi et al [104]	2015	No	Iran	Healthy eating	Health belief model	No
Jing et al [105]	2015	No	China	Healthy eating	Health belief model	No
Jeihooni et al [106]	2015	No	Iran	Calcium intake<br>Vitamin D food sources	Health belief model	No
Gray et al [107]	2015	Yes	USA	Healthy eating	Social cognitive theory<br>Self-determination theory	No
Godinho et al [108]	2015	No	Portugal	Fruits and/or vegetables	Health action process approach	Yes
Frerichs et al [109]	2015	No	USA	Healthy eating	Social cognitive theory	No
Flattum et al [110]	2015	Yes	USA	Healthy eating	Social cognitive theory	No
Duncan et al [111]	2015	No	UK	Fruits and/or vegetables	Theory of planned behaviour and Social cognitive theory	Yes

Cook et al [112]	2015	No	USA	Healthy eating	Social cognitive theory	No
Cameron et al [113]	2015	No	UK	Fruits and/or vegetables<br>Alcohol	Theory of planned behaviour	Yes
Burrows et al [114]	2015	Yes	Australia	Healthy eating<br>Cooking skills	Social cognitive theory	No
Brown et al [115]	2015	No	USA	Fruits and/or vegetables<br>Salt intake	Self-determination theory	No
Taylor et al [116]	2014	Yes	USA	Healthy eating	Transtheoretical model	No
Tavassoli et al [117]	2014	No	Iran	Fruits and/or vegetables	Health belief model	No
Smith et al [118]	2014	No	Australia	Sugar-sweetened beverages	Social cognitive theory<br>Self-determination theory	Yes
Sigman-Grant et al [119]	2014	Yes	USA	Healthy eating	Social learning theory	No
Seth et al [120]	2014	No	USA	Healthy eating	Social cognitive theory	No
Peters et al [121]	2014	Yes	USA	Healthy eating	Social cognitive theory	Yes
Naghashpour et al [122]	2014	Yes	Iran	Calcium intake	Health belief model	No
Muzaffar et al [123]	2014	Yes	USA	Healthy eating	Social cognitive theory	No
Leines et al [124]	2014	Yes	USA	Fruits and/or vegetables	Social cognitive theory	No
Lein et al [125]	2014	Yes	UK	Calcium intake<br>Vitamin D food sources	Health belief model	No
Leblanc et al [126]	2014	Yes	Canada	Mediterranean Diet	Self-determination theory	No
Lana et al [127]	2014	No	Spain and<br>Mexico	Healthy eating &<br>Alcohol	Transtheoretical model	No
Kushida et al [128]	2014	Yes	Japan	Fruits and/or vegetables	Transtheoretical model	No
Kothe et al [129]	2014	No	Australia	Fruits and/or vegetables	Theory of planned behaviour	Yes
**Author (Ref number)**	**Year**	**Dietitian/Nutritionist**	**Country**	**Dietary behaviour**	**Theory**	**BCTs taxonomy**
Kothe et al [130]	2014	No	Australia	Fruits and/or vegetables	Theory of planned behaviour	Yes
Kobel et al [131]	2014	No	Germany	Sugar-sweetened beverages &<br>breakfast skipping	Social cognitive theory	No
Keita et al [132]	2014	Yes	USA	Healthy eating	Social cognitive theory	No
Jancey et al [133]	2014	Yes	Australia	Healthy Eating	Social cognitive theory<br>Transtheoretical model	No
Harmon et al [134]	2014	No	USA	Fruits and/or vegetables<br>Fat intake (decrease)	Social cognitive theory	No
Habib-Mourad et al [135]	2014	Yes	Lebanon	Healthy eating	Social cognitive theory	No
Gold et al [136]	2014	Yes	USA	Food safety knowledge	Theory of planned behaviour	No

Epton et al [137]	2014	No	UK	Fruit and/or vegetables & Alcohol	Theory of planned behaviour	No
Bai et al [138]	2014	Yes	USA	Fruits and/or vegetables	Theory of planned behaviour	No
Annesi et al [139]	2014	No	USA	Healthy eating	Social cognitive theory	No
Yao et al [140]	2013	Yes	USA	Whole grain foods	Social cognitive theory	No
Velicer t al [141]	2013	No	USA	Healthy eating	Transtheoretical model	No
Smith et al [142]	2013	No	USA	Healthy eating	Theory of planned behaviour<br>Social cognitive theory<br>Self-determination theory	No
Ryan et al [143]	2013	No	USA	Calcium intake<br>Vitamin D food sources	Integrated theory of health behavior Change.	No
Rosario et al [144]	2013	Yes	Portugal	Discretionary foods	Health promotion model<br>Social cognitive theory	No
Rani et al [145]	2013	yes	India	Healthy eating	Health belief model	No
Puma et al [146]	2013	Yes	USA	Healthy eating	Social cognitive theory	No
Najimi et al [147]	2013	No	Iran	Fruits and/or vegetables	Social cognitive theory	No
Nabi et al [148]	2013	No	USA	Healthy eating	Social cognitive theory	No
Mead et al [149]	2013	Yes	Canada	Healthy eating	Social cognitive theory<br>Social ecological model	No
Mayurachat et al [150]	2013	No	Thailand	Healthy eating	Theory of planned behaviour	No
Majumdar et al [151]	2013	Yes	USA	Healthy eating	Social cognitive theory<br>Self-determination theory	No
Locher et al [152]	2013	Yes	USA	Healthy eating	Social cognitive theory<br>Social ecological model	No
Linnell et al [153]	2013	Yes	USA	Calcium intake	Social cognitive theory	No
LaBrosse et al [154]	2013	Yes	USA	Folate-rich foods	Health belief model	No
Kerr et al [155]	2013	No	USA	Healthy eating	Social cognitive theory	No
Karimi-Shahanjarini et al [156]	2013	Yes	Iran	Discretionary foods (snacks)	Theory of planned behaviour	No
Hecht et al [157]	2013	Yes	USA	Healthy eating	Theory of planned behaviour	No
**Author (Ref number)**	**Year**	**Dietitian/Nutritionist**	**Country**	**Dietary behaviour**	**Theory**	**BCTs taxonomy**
Gutschall et al [158]	2013	Yes	USA	Healthy eating	Social cognitive theory	No
Gates et al [159]	2013	Yes	Canada	Dairy products	Social cognitive theory	No
Dixon et al [160]	2013	Yes	USA	Healthy eating/Cooking skills	Social cognitive theory	No
Dirige et al [161]	2013	Yes	USA	Fruits and/or vegetables<br>Fat intake	Transtheoretical model	No
Dewar et al [162]	2013	Yes	Australia	Healthy eating	Social cognitive theory	No

Burrows et al [163]	2013	Yes	Australia	Healthy eating and Cooking skills	Social cognitive theory	No
Branscum et al [164]	2013	Yes	USA	Healthy eating	Social cognitive theory	Yes
Bhurosy et al [165]	2013	No	Mauritius	Calcium intake	Health belief model	No
Alexopoulos et al [166]	2013	No	Greece	Lipid intake	Health belief model	No
Aboud et al [167]	2013	No	Bangladesh	Healthy eating	Social learning theory	No
Wenrich et al [168]	2012	Yes	USA	Fruits and/or vegetables	Social cognitive theory	No
Somerville et al [169]	2012	Yes	USA	Fruits and/or vegetables	Social cognitive theory	No
Poddar et al [170]	2012	Yes	USA	Dairy products	Social cognitive theory	No
Lubans et al [171]	2012	Yes	Australia	Healthy eating	Social cognitive theory	No
Lent et al [172]	2012	Yes	USA	Healthy eating	Social cognitive theory	No
Kothe et al [173]	2012	No	Australia	Fruits and/or vegetables	Theory of planned behaviour	Yes
Horodynski et al [174]	2012	Yes	USA	Healthy eating	Social cognitive theory	No
Breslin et al [175] [176]	2012	Yes	UK	Healthy eating	Social cognitive theory	No
Beaulieu et al [177]	2012	No	Canada	Discretionary foods (takeaway)	Theory of planned behaviour	No
Winett et al [178]	2011	No	USA	Healthy eating	Social cognitive theory	No
Smith et al [179]	2011	No	USA	Healthy eating	Social cognitive theory<br>Self-determination theory<br>Theory of planned behaviour	No
Sharma et al [180]	2011	Yes	USA	Healthy eating	Social cognitive theory	No
Seal et al [181]	2011	No	USA	Healthy eating	Social cognitive theory	No
Salehi et al [182]	2011	No	Iran	Fruits and/or vegetables	Transtheoretical model	No
Prelip et al [183]	2011	No	USA	Fruits and/or vegetables	Social cognitive theory<br>Theory of planned behaviour	No
Lv et al [184]	2011	Yes	USA	Calcium intake and vitamin D	Theory of planned behaviour	No
Kothe et al [185]	2011	No	Australia	Breakfast consumption	Theory of planned behaviour	No
Jung et al [186]	2011	No	Canada	Calcium intake	Health belief model	No
Jones et al [187]	2011	Yes	USA	Healthy eating	Social cognitive theory	No
Jemmott et al [188]	2011	No	South African	Healthy eating	Social cognitive theory<br>Theory of planned behaviour	No
Hendrie et al [189]	2011	Yes	Australia	Dairy products	Social learning theory	No
Chen et al [190]	2011	No	USA	Healthy eating	Transtheoretical model<br>Social cognitive theory	No
**Author (Ref number)**	**Year**	**Dietitian/Nutritionist**	**Country**	**Dietary behaviour**	**Theory**	**BCTs taxonomy**
Bukhari et al [191]	2011	Yes	USA	Healthy eating	Social cognitive theory<br>Social ecological model	No

Abi Haidar et al [192]	2011	Yes	Lebanon	Water consumption	Health belief model	No
Zhou et al [193]	2010	No	China	Healthy eating	Transtheoretical model	No
Zeinstra et al [194]	2010	Yes	Netherlands	Fruits and/or vegetables	Self-determination theory	No
Walker et al [195]	2010	Yes	USA	Healthy eating	Health promotion model	No
Poddar et al [196]	2010	Yes	USA	Dairy products	Social cognitive theory	No
Pearson et al [197]	2010	No	UK	Fruits and/or vegetables	Social cognitive theory	No
Milan et al [198]	2010	Yes	USA	Folate supplements	Transtheoretical model	No
Irwin et al [199]	2010	No	USA	Healthy eating	Social cognitive theory	No
Freedman et al [200]	2010	Yes	USA	Healthy eating	Social cognitive theory	No
Freedman et al [201]	2010	Yes	USA	Healthy eating	Social cognitive theory	No
Contento et al [202]	2010	Yes	USA	Healthy eating	Social cognitive theory<br>Self-determination theory	No
Brown et al [203]	2010	Yes	USA	Healthy eating	Social cognitive theory	No
Black et al [204]	2010	Yes	USA	Healthy eating	Social cognitive theory	No
Topp et al [205]	2009	No	USA	Healthy eating	Transtheoretical model	No
Shilts et al [206]	2009	Yes	USA	Healthy eating	Social cognitive theory	No
Rosenkranz et al [207]	2009	Yes	USA	Healthy eating	Social cognitive theory	No
Peng et al [208]	2009	No	USA	Healthy eating	Health belief model<br>Social cognitive theory<br>Theory of reasoned action	No
Lubans et al [209]	2009	Yes	Australia	Healthy eating	Social cognitive theory	No
Koning et al [210]	2009	No	Netherlands	Alcohol consumption	Theory of planned behaviour<br>Social cognitive theory	No
Gans et al [211]	2009	No	USA	Fruits and/or vegetables<br>Fat intake	Transtheoretical model<br>Social cognitive theory	No
Elder et al [212]	2009	No	USA	Fibre intake<br>Fat intake<br>Fruits and/or vegetables	Social cognitive theory	No
Clifford et al [213]	2009	Yes	USA	Fruits and/or vegetables	Social cognitive theory	No
Clark et al [214]	2009	Yes	USA	Infant feeding practices	Social learning theory	No
Branscum et al [215]	2009	Yes	USA	Healthy eating	Social cognitive theory	No
Angelopoulos et al [216]	2009	Yes	Greece	Healthy eating	Theory of planned behaviour	No
Zepeda et al [217]	2008	Yes	USA	Healthy eating	Precaution adoption process model	No
Resnicow et al [218]	2008	No	USA	Fruits and/or vegetables	Self-determination theory	No
Park et al [219]	2008	Yes	USA	Fruits and/or vegetables	Transtheoretical model	No

Newman et al [220]	2008	Yes	USA	Healthy eating	Social cognitive theory	No
**Author (Ref number)**	**Year**	**Dietitian/Nutritionist**	**Country**	**Dietary behaviour**	**Theory**	**BCTs taxonomy**
Kroeze et al [221]	2008	Yes	Netherlands	Fat intake	Precaution adoption process model<br>Theory of planned behaviour	No
Kelley et al [222]	2008	Yes	USA	Healthy eating	Theory of planned behaviour	No
Ince et al [223]	2008	No	Turkey	Healthy eating	Social cognitive theory	No
Greene et al [224]	2008	Yes	USA	Fruits and/or vegetables	Transtheoretical model	No
Di Noia et al [225]	2008	Yes	USA	Fruits and/or vegetables	Transtheoretical model	No
Canavera et al [226]	2008	Yes	USA	Fruits and/or vegetables<br>Sugar-sweetened beverages	Social cognitive theory	No
Burgess-Champoux et al [227]	2008	Yes	USA	Whole grain foods	Social cognitive theory	No
Bohaty et al [228]	2008	No	USA	Calcium intake<br>Vitamin D food sources	Self-efficacy theory	No
Ahmed [229]	2008	No	Egypt	Breastfeeding Knowledge	Social cognitive theory	No
Nitzke et al [230]	2007	Yes	USA	Fruits and/or vegetables	Transtheoretical model	No
Manios et al [231]	2007	Yes	Greece	Calcium intake<br>Vitamin D food sources	Health belief model<br>Social cognitive theory	No
Lautenschlager et al [232]	2007	Yes	USA	Healthy eating	Theory of planned behaviour	No
Plotnikoff et al [233]	2005	Yes	Canada	Healthy eating	Social cognitive theory<br>Transtheoretical Model<br>Protection motivation theory<br>Theory of Planned Behavior.	No
Gratton et al [234]	2007	No	UK	Fruits and/or vegetables	Theory of planned behaviour	No
Doerksen et al [235]	2007	No	USA	Fruits and/or vegetables	Social cognitive theory	No
Contento et al [236]	2007	Yes	USA	Healthy eating	Theory of planned behaviour	No
Colby et al [237]	2007	Yes	USA	Healthy eating	Social ecological model<br>Social cognitive theory	No
Armitage et al [238]	2007	No	UK	Fruits and/or vegetables	Theory of planned behaviour	No
Evans et al [239]	2006	Yes	USA	Healthy eating	Social cognitive theory	No
Tussing et al [240]	2005	Yes	USA	Calcium intake	Health belief model<br>Theory of reasoned action	No
Tsorbatzoudis et al [241]	2005	No	Greece	Healthy eating	Theory of planned behaviour	No
Schrader et al [242]	2005	No	USA	Calcium intake	Health belief model	No
Powers et al [243]	2005	Yes	USA	Healthy eating	Social cognitive theory	No
Frenn et al [244]	2005	No	USA	Fat intake	Transtheoretical model	No

Author (Ref number)	Year	Dietitian/Nutritionist	Country	Dietary behaviour	Theory	BCTs taxonomy
Cullen et al [245] Baranowski [264]	2005	Yes	USA	Fruits and/or vegetables	Social cognitive theory	No
Anderson et al [246]	2005	Yes	UK	Fruits and/or vegetables	Theory of planned behaviour	No
Rinderknecht et al [247]	2004	Yes	USA	Healthy eating	Social cognitive theory	No
Lytle et al [248]	2004	Yes	USA	Fibre intake<br>Fat intake	Social cognitive theory	No
Levy et al [249]	2004	Yes	USA	Cooking skills for Healthy eating	Social learning theory	No
Helinski et al [250]	2004	Yes	USA	Folic-acid intake	Health belief model	No
Carpenter et al [251]	2004	Yes	USA	Healthy eating	Social cognitive theory<br>Transtheoretical model	No
Campbell et al [252]	2004	Yes	USA	Fruits and/or vegetables<br>Fat intake	Social cognitive theory<br>Transtheoretical model<br>Health belief model	No
Campbell et al [253]	2004	Yes	USA	Healthy eating	Social cognitive theory<br>Transtheoretical model	No
Abood et al [254]	2004	No	USA	Healthy eating	Social cognitive theory	No
Veverka et al [255]	2003	Yes	USA	Healthy eating	Transtheoretical model	No
Van Horn et al [256]	2003	Yes	USA	Fat intake	Social cognitive theory<br>Social Action Theory	No
Steptoe et al [257]	2003	No	UK	Fruits and/or vegetables	Social learning theory and Stage of Change ie Transtheoretical model	No
Holmes et al [258]	2003	Yes	USA	Folic-acid enriched foods	Social cognitive theory	No
Gribble et al [259]	2003	Yes	USA	Fruits and/or vegetables	Social learning theory	No
Gaughan et al [260]	2003	Yes	USA	Healthy eating	Social cognitive theory	No
Frenn et al [261]	2003	No	USA	Fat intake	Transtheoretical model<br>Health promotion model	No
Derrickson et al [262]	2003	Yes	USA	Healthy eating	Social learning theory<br>Self-efficacy theory	No
Davis et al [263]	2003	Yes	USA	Healthy eating	Social learning theory	No
Abood et al [265]	2003	Yes	USA	Healthy eating	Health belief model	No
Wilson et al [266]	2002	No	USA	Fruits and/or vegetables	Social cognitive theory	No
Morris et al [267]	2002	Yes	USA	Fruits and/or vegetables	Social cognitive theory	No
Evans et al [268]	2002	Yes	USA	Healthy eating	Social cognitive theory	No

Schnoll et al [269]	2001	Yes	USA	Fibre intake	Social cognitive theory	No
Anderson et al [270]	2001	No	USA	Healthy eating	Social cognitive theory	No
Siero et al [271]	2000	No	Netherlands	Mediterranean Diet	Transtheoretical model	No
Macpherson et al [272]	2000	Yes	USA	Healthy eating	Social learning theory	No
Havas et al [273]	2000	Yes	USA	Fruits and/or vegetables	Transtheoretical model	No
Gray-Donald et al [274]	2000	Yes	Canada	Healthy eating	Social learning theory	No
Finckenor et al [275]	2000	Yes	USA	Fat intake	Transtheoretical model	No
Baranowski et al [276]	2000	Yes	USA	Fruits and/or vegetables	Social cognitive theory	No
